# Experiencing neutropenia: Quality of life interviews with adult cancer patients

**DOI:** 10.1186/1472-6955-4-4

**Published:** 2005-07-08

**Authors:** Barry V Fortner, Kurt W Tauer, Ted Okon, Arthur C Houts, Lee S Schwartzberg

**Affiliations:** 1 West Clinic, 100 Humphreys Blvd Suite 100, Memphis, TN 38104 USA; 2 Supportive Oncology Services and Accelerated Community Oncology Research Network, 1790 Kirby Parkway, Suite 101, Memphis, TN 38138 USA

## Abstract

**Background:**

Neutropenia is a common toxicity in chemotherapy but detailed information about how neutropenia is associated with changes in patients' quality of life is not readily available. This prospective study interviewed patients with grade 4 neutropenia to provide qualitative information on patients' experience of developing and coping with grade 4 neutropenia during a cycle of chemotherapy.

**Methods:**

A sample of 34 patients who developed grade 4 neutropenia during the first cycle of chemotherapy completed a total of 100 structured clinical interviews. Interviews were transcribed, and 2 raters inductively developed 5 broad categories comprising 80 specific complaint domains nominated by patients. Thirty-five patient-nominated problems were mentioned in 5% or more of the interviews.

**Results:**

Fatigue was the most common physical symptom. Interference in daily routine, negative self-evaluation, negative emotion, and social isolation were other common complaints associated with neutropenia.

**Conclusion:**

Neutropenia is associated with a number of negative experiences among cancer patients undergoing chemotherapy, and these negative experiences have an adverse effect on the patient's quality of life. Oncology nurses can play a key role in helping patients manage adverse effects to maintain their quality of life.

## Background

Quality of life (QOL) issues have historically played an important part in the nursing role of patient advocacy. Ropka et al. found in the Year 2000 ONS Research Priorities Survey that quality of life ranked second among the top 20 research priorities of sampled nurses [1]. Other priorities included neutropenia/immunosuppression (fifth), as well as depression and stress-coping adaptation in thirteenth and fourteenth place respectively. These research priorities and the realization that accurate assessment of QOL in patients can provide both physicians and nurses with important clinical information [2] have provided the framework for initiating this research on the correlation between chemotherapy-induced neutropenia and QOL.

The term quality of life has been defined and measured in many different ways in the literature. Haas [3] offers the following definition to differentiate QOL from closely related concepts such as well-being, satisfaction with life, and functional status:

"QOL is a multidimensional evaluation of an individual's current life circumstances in the context of the culture and value systems in which they live and the values they hold. QOL is primarily a subjective sense of well-being encompassing physical, psychological, social and spiritual dimensions..."

Complications and side effects of cancer treatment can not only adversely affect clinical outcomes, but also negative influence a patient's quality of life. Chemotherapy specifically can impact the patient's QOL by significantly impacting social, physical and global functionality [4].

Many cancer chemotherapies work by suppressing fast-dividing cells like cancer cells; however, the fast-dividing cells of the bone marrow, which are responsible for producing the cells involved in the first line immune defense such as neutrophils and other white cells, can also be negatively affected. Neutropenia, i.e., decreased white blood cell counts, is the name given to this side effect of such myelosuppressive chemotherapies. The National Cancer Institute's Common Toxicity Criteria provides for 5 grades of neutropenia (Grade 5 = death) [5]. Grade 4 neutropenia is defined by an absolute neutrophil count (ANC) of less than 0.5 × 10^9 ^cells/L, commonly referred to as an ANC of less than 500 cells/mL. By comparison, grade 0, which is considered a normal count, varies from 3 to 6 × 10^9 ^cells/L. When patients develop febrile neutropenia (grade 4 neutropenia plus fever [i.e., a body temperature of greater than 38.5°C), they are often hospitalized and placed on antibiotics to prevent life-threatening sepsis [6,7]. In addition to the clinical consequences of febrile neutropenia, hospitalization and possible isolation can have a significant impact on a patient's quality of life.

Little is actually known about the impact of neutropenia on quality of life, which has only occasionally been examined as a secondary outcome in the context of side effects of chemotherapies. Fortner [8] reported on a cohort of 62 patients receiving chemotherapy with no growth factors. Patients completed different scales (SF-36, Cancer Care Monitor, Psychosocial Adjustment to Illness Scale (PAIS)) at weekly intervals before having their absolute neutrophil count (ANC) measured. The results of this study provided preliminary evidence of the association between CIN and QOL impairments.

Oncology nurses are increasingly playing a major role in the management of neutropenia. Recent nursing publications have reported on the suitability of the nurse, as a direct caregiver, to assess patients' potential risk factors for neutropenia and implement guidelines for management [9,10]. Maxwell et al [11] showed how the utilization guidelines for neutropenia management could not only save nursing staff considerable time, but also improve the overall flow of patient care for clinical practices. An increased knowledge of how CIN affects QOL would assist nurses in their patient education efforts and allow them to direct patients' preventative care measures during myelosuppressive chemotherapy regimens.

The purpose of this preliminary investigation was to obtain detailed information from patients, describing the experience of becoming neutropenic. In particular, by conducting qualitative interviews during neutropenic periods, patients were assessed on the impact it had on their quality of life and their routine functioning. Whereas we value and have, in fact, deployed standardized, quantitative measures of quality of life, this report focuses on qualitative information. Such qualitative information is often obtained to assist in the development of more finely-tuned, quantitative measures of a given condition. The pilot study reported here has resulted in a prospective study, results of which are going to be reported in the near future.

## Methods

### Design and participants

A convenience sample of adult cancer patients with grade 4 neutropenia was evaluated in the pilot study to determine the impact of neutropenia on patient psychological adjustment and functioning. The prospective design restricted participation to adult cancer patients from the West Clinic (Memphis, Tennessee) who were scheduled to receive the first cycle of one of the following chemotherapy regimens, at the full dose, given in either a 21- to 28-day cycle: (1) docetaxel, (2) AT (doxorubicin and paclitaxel), (3) CHOP (cyclophosphamide, doxorubicin, vincristine, and prednisone), (4) ESHAP (etoposide, methylprednisolone, cisplatin, and cytarabine), (5) carboplatin-paclitaxel, (6) carboplatin-docetaxel, and (7) carboplatin-gemcitabine. These chemotherapies were selected because they were judged to be among those that carried a significant risk of producing grade 4 neutropenia [7,12-15]. There was no restriction on cancer type. Patients were excluded if they were already participating in another formal clinical trial; were scheduled to receive supportive treatment with colony-stimulating factors (eg, filgrastim or sargramostim); or had a life expectancy less than three months.

Patients visited the clinic on days 0, 7, 14, and 21 of the chemotherapy cycle for assessment of vital signs, complete blood count (CBC) with differential, and QOL assessment. For this analysis, 34 patients who developed grade 4 neutropenia (ANC < 0.5 × 10^9^/L) at any point during their first cycle of chemotherapy were interviewed and completed a total of 100 interviews. Demographic and medical characteristics of the 34 participants are shown in Tables 1 and 2. Seventy percent or more were white, married women who had high school or greater level of education. Consistent with first cycle treatment in an outpatient setting, most participants had a good (0 to 1) Eastern Collaborative Oncology Group (ECOG) performance rating [16]. Over two thirds of patients received docetaxel, and nearly half of the patients had either breast cancer or lung cancer. Only 5 of the 34 patients that were interviewed exhibited a significant fever (≥101.4°F) that required hospitalization.

**Table 1:** Demographic Characteristics

Number of Patients with Grade 4 Neutropenia	34
Mean age in years (range)	61.4 (27 – 76)
Women	71%
Ethnic background	
Black/African American	18%
White/not Hispanic	82%
Education ≥12 years	76%
Marital status	
Married	70%
Single	6%
Divorced	3%
Widowed	21%
Family income ($)	
Under 14,600	11%
14,601 to 29,999	19%
30,000 to 49,999	18%
50,000 to 79,999	29%
80,000 to 99,999	15%
100,000 to 220,000	8%

**Table 2:** Medical Characteristics of 34 Adult Cancer Patients with Grade 4 Neutropenia

Good ECOG performance status^a^	79%
Cancer diagnosis	
Breast	28%
Lung	24%
Lymphoma	15%
Ovarian	9%
Prostate	6%
Other diagnoses	18%
Metastatic disease	37%
Type of chemotherapy	
Docetaxel	70%
CHOP	12%
Carboplatin-docetaxel	15%
Carboplatin-gemcitabine	3%
Hospitalization due to neutropenia and grade 3 fever	15%

^a ^Good performance status is defined as ECOG performance scale score of 0 to 1 on a 5-point scale, where 0 is fully active, and 4 is completely disabled [13].

### Procedure

Participants completed informed consent procedures approved by Western Institutional Review Board (Olympia, Washington). Participants were compensated for their participation.

#### Interview schedule

Participants were scheduled to be interviewed on days 7, 10, 14, and 21 of their respective chemotherapy cycle, but the interviews only began once a patient had an ANC < 0.5 × 10^9^/L (grade 4). Nursing staff in consultation with treating physicians examined the laboratory results and contacted research staff to come and interview the patient. For example, a patient who first developed grade 2 neutropenia on day 7 and grade 4 neutropenia on day 10 would have contributed only 3 interviews (days 10, 14, and 21). Thirty-three of the 34 patients who developed grade 4 neutropenia did so on or before day 10 of chemotherapy; one patient who was receiving carboplatin-gemcitabine chemotherapy first developed grade 4 neutropenia on day 21.

#### Interview protocol

The interviews were conducted by trained interviewers during clinic visits. Interviewers were 4 advanced graduate students in clinical psychology who were highly trained and experienced in structured clinical interviewing techniques with special emphasis on employing empathic reflection, asking open-ended questions, and using techniques to promote patient elaboration of their own experiences. These interviewers were part of the clinics staff in clinical research assistant positions.

The interview protocol was designed to obtain the participant's perspective of the impact of neutropenia within the context of an empathic conversation. Although not administered in this study, the interviewers had also been trained in the Structured Clinical Interview to Diagnose DSM-IV Axis I Disorders (SCID) and were familiar with the criteria needed to identify individuals who might need special assistance and referral for severe psychiatric difficulties [17].

Medical staff (either physicians or nurses) informed participants of their current ANC and, if appropriate, explained that the condition was called neutropenia. Patients were instructed in how to keep a diary of symptoms and how to monitor temperature with a thermometer that was provided for home use. Patients were then interviewed by the clinical assistant. Interviews lasted 20 minutes and were retrospective accounts of the impact of neutropenia covering the period between the interview and the preceding visit. The following topics were covered, and all were phrased in terms of changes since the previous visit: physical feelings and sensations, reduction and/or cessation of activities, interactions with others, financial impacts, ability to work, sex life, emotions, satisfaction with medical care, thoughts about disease, thoughts about treatment procedures, and overall quality of life. These areas were covered because they represent aspects of quality of life that might be affected by grade 4 neutropenia. In addition, interviewers encouraged participants to talk freely and to describe in their own words how they were feeling and how their daily living had been different after developing neutropenia. Every interview was recorded and transcribed verbatim.

### Preliminary analyses

Two raters characterized the information in the interviews by reviewing all transcripts and inductively developing descriptive categories for the nature of patient concerns expressed in the transcript [18-20]. Raters resolved discrepancies by mutual agreement. This procedure yielded 80 unique, descriptive problem domains, distributed over 5 broad categories as follows: Physical Complaints (n = 29), Daily Routine Disruptions (n = 14), Negative Thought and Self Evaluation (n = 17), Negative Emotions (n = 10), and Social Relationships (n = 10). Two raters then counted the instances of each problem domain within the 100 interview transcripts, and any discrepancies were resolved by mutual agreement. Table 3 illustrates the 5 broad categories along with selected unique problem domains and an illustrative exemplar for the displayed problem domain.

**Table 3:** Categories and Exemplars of Patient-nominated Effects of Neutropenia

Major Category Specific Problem Domain	Illustrative Exemplars in Patients' Own Words
Physical Complaints	
Tired, fatigued, exhausted, weak, feeling "drained"	If I walk from one room to the next, I'm worn out.
Reduced sexual activity	It has definitely slowed down. I don't have a great deal of interest.
Daily Routine Disruptions	
Loss of daily routine	I have stopped going to church and taking my son to school.
Difficulties with household activities	I have stopped most everything. I am not doing my housework.
Negative Thought and Self Evaluation	
Worrying about paying for extra care needed	If I don't work, I don't get paid. It's getting very scary.
Feeling useless/helpless, letting people down	I feel guilty when I see other people doing work I should be doing.
Negative Emotions	
Feeling down	I feel like giving up. I just wish it would all end.
Feeling anxious	I am scared. I hope I don't have to go through this (neutropenia) the next three times I come here.
Social Relationships	
Decreased social contacts and activity	I can't spend time with other people because I am sick.
Avoiding crowds	I don't go to church because someone might hug me or sneeze near me.

Note: This table contains selected exemplars of the problem domains presented by patients in the five broad categories identified as follows: Physical Complaints, Daily Routine Disruptions, Negative Thought and Self Evaluation, Negative Emotions, and Social Relationships.

## Results

Tables 4 through 8 illustrate the results of the tabulations of only the unique problem domains that were nominated in 5% or more of the interview. This restriction permitted a balance between rich description and characteristic or representative patient reactions to grade 4 neutropenia. These tables are designed to show how often a given problem was mentioned within the 100 interviews and also how frequently an individual patient mentioned the given problem, and data are presented in order of descending frequency. Altogether, the tables present information about 35 of the 80 specific problem domains identified in the content analysis of the interview transcripts.

**Table 4:** Patient-nominated Physical Complaints During and After Grade 4 Episodes of Chemotherapy-induced Neutropenia

Patient-nominated Physical Complaint	Frequency Per Interview (N = 100) n (%)	Frequency Per Patient (N = 34) n (%)
Tired, fatigued, exhausted, weak, feeling "drained"	69 (69%)	31 (91%)
Sleep interruption (eg, onset insomnia, waking)	19 (19%)	14 (41%)
Muscle aches, feeling achy, swollen joints	16 (16%)	13 (38%)
Pain	15 (15%)	10 (29%)
Cough, sore throat, mucous in throat, problems swallowing	10 (10%)	8 (24%)
Reduced sexual activity (eg, too tired for sex)	9 (9%)	6 (18%)
Change in taste of foods	7 (7%)	6 (18%)
Decreased appetite	7 (7%)	7 (21%)
Diarrhea	7 (7%)	6 (18%)
Stomach upset, cramping	7 (7%)	7 (21%)
Mouth Sores	6 (6%)	6 (18%)
Nausea	5 (5%)	5 (15%)
Skin problems (rash, skin breaking out, dry skin)	5 (5%)	3 (9%)

Note: Thirty-four patients completed 100 interviews with each patient contributing between 1 and 4 interviews. To be included in the table, the complaint had to have been nominated within at least 5% of the interviews.

**Table 8:** Patient-nominated Social Relationship Changes During and After Grade 4 Episodes of Chemotherapy-induced Neutropenia

Patient-nominated Social Relationship Changes	Frequency Per Interview (N = 100) n (%)	Frequency Per Patient (N = 34) n (%)
Decreased social contacts and activity	38 (38%)	20 (59%)
Avoiding crowds	17 (17%)	11 (32%)
Other people getting on my nerves	10 (10%)	8 (24%)
Feeling lonely and isolated	6 (6%)	6 (18%)
Withdrawing and keeping to myself	6 (6%)	4 (12%)

Note: Thirty-four patients completed 100 interviews with each patient contributing between 1 and 4 interviews. To be included in the table, the complaint had to have been nominated within at least 5% of the interviews.

Table 4 shows the frequency of the occurrence of patient-nominated problem domains broadly categorized as physical complaints. The most frequent problem mentioned by these patients with grade 4 neutropenia was fatigue, which was expressed using terms such as feeling tired, weak, and drained. Fully 91% of the participants complained about fatigue and its interference with their daily lives. In terms of frequency in the interviews, no other physical complaint matched fatigue, which was a complaint in nearly 70% of the interviews compared with less than 20% for other problems across interviews. Sleep interruption, muscle aches, pain, and sore throat were the next most frequently mentioned physical complaints, and these affected between 41% and 14% of the patients interviewed. With the exception of skin irritations, which affected 9% of the patients, the remaining problems listed in Table 4 affected between 15% and 21% of the participants.

It should be noted that, in part because of the nature of the data, while we have an association between grade 4 neutropenia and these complaints, we do not have a causal link. It is entirely likely that some of these complaints such as fatigue and exhaustion are endemic to chemotherapy and would occur at a comparable rate among similar patients with similar cancers undergoing the same chemotherapy, only without the condition of grade 4 neutropenia. Given what little is known about the experience of neutropenia from the patient's perspective, this may be a reasonable start to finding out how the physical impact of neutropenia affects patient functioning and quality of life. The top five complaints listed in Table 4 certainly could be complaints that are exacerbated, if not caused, by neutropenia.

Table 5 lists the most frequently mentioned effects of neutropenia on patients' daily routine. Over half of the patients noted that their daily routine was interrupted, preventing them from leading normal lives. This effect was especially noted in terms of physical activity and may simply be a correlate of the severe fatigue that nearly all of the patients reported. In terms of quality of life, this interference with the activities of daily living prevented these neutropenic patients from engaging in the physical and social activities that brought them pleasure and made life enjoyable.

**Table 5:** Patient-nominated Daily Routine Disruption Problems During and After Grade 4 Episodes of Chemotherapy-induced Neutropenia

Patient-nominated Daily Routine Disruption Problems	Frequency Per Interview (N = 100) n (%)	Frequency Per Patient (N = 34) n (%)
Loss of daily routine ("Cannot live life like I used to")	42 (42%)	19 (56%)
Unable to engage in normal physical activities	39 (39%)	19 (56%)
Difficulties with household activities (eg cooking)	34 (34%)	19 (56%)
Unable to work like before	27 (27%)	13 (38%)
Laying around more and napping	25 (22%)	18 (53%)
Unable to engage in normal non-physical hobbies	23 (23%)	16 (47%)
Unable to attend religious services	21 (21%)	12 (35%)
Difficulties with childcare	9 (9%)	6 (18 %)

Note: Thirty-four patients completed 100 interviews with each patient contributing between 1 and 4 interviews. To be included in the table, the complaint had to have been nominated within at least 5% of the interviews.

Table 6 covers the broad category of negative thinking and negative self-evaluation. The experience of becoming neutropenic was associated with a sense of dread and increased fear about continuing further chemotherapy treatment for nearly one fourth of these patients. Interviewers were struck by the fact that some patients seemed to begin to question their own self worth as they became increasingly isolated and restricted because of lack of energy and the necessary medical prohibitions to avoid social contact during the period of high risk for infection (ie, severe neutropenia), even though this type of complaint was less frequent than physical complaints like fatigue. This negative self-evaluation was evident in approximately 1 out of 5 patients who developed grade 4 neutropenia.

**Table 6:** Patient-nominated Negative Thought and Self Evaluation Changes During and After Grade 4 Episodes of Chemotherapy-induced Neutropenia

Patient-nominated Negative Thought and Self Evaluation Changes	Frequency Per Interview (N = 100) n (%)	Frequency Per Patient (N = 34) n (%)
Feeling of dread about coming for chemotherapy	10 (10%)	9 (27%)
Worrying about paying for extra care needed	9 (9%)	5 (15%)
Feeling useless/helpless, letting people down	7 (7%)	6 (18%)
Sense of loss of independence	6 (6%)	5 (15%)
Worrying about being around other sick people	5 (5%)	5 (15%)

Note: Thirty-four patients completed 100 interviews with each patient contributing between 1 and 4 interviews. To be included in the table, the complaint had to have been nominated within at least 5% of the interviews.

Table 7 details the most frequent negative emotions mentioned during interviews. Not surprisingly, nearly one fourth of the patients complained that they felt down or blue. This may be a natural consequence of the fact that over half of all the participants reported a restriction in their daily activities that brought them pleasure and a sense of accomplishment. A bit more surprising was the fact that over 20% of participants reported feeling more irritable and short tempered after episodes of neutropenia.

**Table 7:** Patient-nominated Negative Emotions During and After Grade 4 Episodes of Chemotherapy-induced Neutropenia

Patient-nominated Negative Emotions	Frequency Per Interview (N = 100) n (%)	Frequency Per Patient (N = 34) n (%)
Feeling down	21 (21%)	8 (24%)
Feeling irritable and frustrated	12 (12%)	7 (21%)
Crying more than usual	5 (5%)	4 (12%)
Feeling anxious	5 (5%)	5 (15%)

Note: Thirty-four patients completed 100 interviews with each patient contributing between 1 and 4 interviews. To be included in the table, the complaint had to have been nominated within at least 5% of the interviews.

Table 8 illustrates social relationship changes associated with grade 4 neutropenia. Most patients (59%) reported a decrease in social contacts and social outings. This most likely resulted from the combination of the reported severe fatigue and the doctors' orders to refrain from social contact during the period of high infection risk. This experience of being isolated from customary social contacts and activities was uniformly experienced as a negative aspect of neutropenia.

## Conclusion

The picture of neutropenia that emerges from these structured clinical interviews with grade 4 neutropenic cancer patients undergoing chemotherapy illustrates their diminished quality of life, wherein fatigue, negative emotion, and a sense of isolation and reduced self worth are featured. To be sure, not every patient studied experienced the full spectrum of these problems, and, even among those who did, the intensity of the experience varied from mild to rather severe, especially for the 5 individuals who were hospitalized with fever. Although neutropenia is characterized by the lab value ANC, neutropenia clearly has a broader consequence, considering how it can impact the quality of life of the affected patient and potentially endanger life. Results from these interviews may be useful in constructing measures of quality of life outcomes in chemotherapy patients who develop neutropenia. Physical symptoms are clearly important for any such measure, and inclusion of some measure of fatigue or exhaustion is mandatory. As one might expect from compromised immune functioning, patients mentioned some obvious physical symptoms such as sore throat, mouth sores, and swollen joints. Less intuitively obvious, but nevertheless important, were other symptoms such as sleep interruption and reduced sexual activity. These suggest that even the physical symptoms associated with neutropenia are not as straightforward as one might expect. The impact of neutropenia goes beyond physical symptoms to affect social, cognitive, and emotional functioning of patients.

We found that most patients reported significant disruptions to their daily routine. The degree to which their fatigue and exhaustion interfered with their ability to live a normal life was striking. Instead of being able to carry on as before, these patients reported that they were upset and often felt guilty about not being able to do those routine tasks that gave them a sense of personal fulfillment and pleasure. Being in this debilitated state and unable to carry on with their usual routines also led to negative self-appraisals and negative emotions. It should be noted that we do not regard these reactions to be unusual or out of the ordinary. In fact, we consider these reactions to be normal, considering the very difficult physical conditions of undergoing chemotherapy with the added burden of developing neutropenia and the fact that we did not observe psychiatric disorders among these patients.

Continued research is needed to establish specific correlations between physical symptoms caused by neutropenia and their psychosocial effects. This will further assist nurses and physicians to understand the impact of neutropenia on patients and allow them to institute specific interventions to decrease the severity of side effects from myelosuppresive chemotherapy as well as to ameliorate their influence on the patients' quality of life. As a qualitative and preliminary study of the quality of life implications of neutropenia, this investigation has some important limitations. We focused on those patients who became neutropenic as defined by grade 4 neutropenia. Future research should investigate the human qualitative impact of lesser grades of neutropenia. We began with this extreme group to get as clear a picture as possible for this condition. Furthermore, it is also important to discover how these difficulties develop as patients progress from a normal ANC through various grades of neutropenia. For example, there may be subjectively reported precursors of grade 4 neutropenia that patients can identify when they have grade 3 neutropenia. This might be clinically important to facilitate identifying those patients who will be at greater risk for developing severe neutropenia that may require hospitalization.

Another important limitation of this investigation is the lack of a comparison group, e.g., patients who were receiving chemotherapy but who did not experience significant neutropenia. When one looks at the content of the complaints that patients presented in this investigation, some of those seem like they would be complaints likely to surface for any patient undergoing chemotherapy. For example, a change in the taste perception and nausea are unlikely to be unique to neutropenia, and may reflect common complaints for a range of chemotherapy patients. Considering the major physical complaints of fatigue and a sense of exhaustion, it would be important to determine to what extent that complaint was generally true of chemotherapy patients versus being a complaint unique to, or especially exacerbated among, chemotherapy patients who developed significant neutropenia. It should also be noted that in this prospective study we did not attempt to correlate additional laboratory values such as hemoglobin that might be related to fatigue associated with anemia. Such important factors should be addressed in more controlled studies where patients with particular cancers undergoing specific chemotherapy regimens can be followed prospectively to make the appropriate comparison. In addition, experimental investigations where patients are randomly assigned to receive neutropenia preventive interventions such as colony stimulating factors could help to clarify the extent to which such fatigue is caused by neutropenia as distinct from caused by chemotherapy alone.

Neutropenia is certainly a serious medical condition that many cancer patients will have to face. This has been known for quite some time. What has been less obvious is that neutropenia is a medical condition with widespread cognitive, emotional, and social impacts for patients. In addition to developing treatment protocols for preventing and treating neutropenia from a medical standpoint, it is also important to consider the human experience side from the point of view of the patient. Both febrile and afebrile neutropenia present formidable challenges to chemotherapy patients, and their caregivers need to be aware of these challenges to design treatments that adequately address them.

## Competing interests

Funding for this research was provided by Supportive Oncology Services, Ted Okon, CEO, and by an unrestricted educational grant from Amgen Inc. (Thousand Oaks, California). Amgen is the manufacturer of Neulasta^® ^(pegfilgrastim) and NEUPOGEN^® ^(Filgrastim), indicated for reducing the incidence of infection associated with chemotherapy-induced neutropenia in cancer patients with nonmyeloid malignancies.

## Authors' contributions

BF conceived of the study and participated in the design and coordination of the study. KT participated in the design and coordination of the study. TO participated in the coordination of the study and draft of the manuscript. AH participated in the design of the study, conducted the summary of data findings and drafted the manuscript. LS participated in the design of the study and development of the study protocol. All authors read and approved the final manuscript.

## Pre-publication history

The pre-publication history for this paper can be accessed here:


